# Heterologous Expression of Three Transcription Factors Differently Regulated Astragalosides Metabolic Biosynthesis in *Astragalus membranaceus* Hairy Roots

**DOI:** 10.3390/plants11141897

**Published:** 2022-07-21

**Authors:** Xiao Hua Li, Jae Kwang Kim, Sang Un Park

**Affiliations:** 1 School of Life Science and Technology, Wuhan Polytechnic University, Wuhan 430023, China; lixiaohua20212021@163.com; 2 Convergence Research Center for Insect Vectors, Division of Life Sciences, College of Life Sciences and Bioengineering, Incheon National University, 119 Academy-ro, Yeonsu-gu, Incheon 22012, Korea; 3 Department of Crop Science, Chungnam National University, 99 Daehak-ro, Yuseong-gu, Daejeon 34134, Korea; 4 Department of Smart Agriculture Systems, Chungnam National University, 99 Daehak-ro, Yuseong-gu, Daejeon 34134, Korea

**Keywords:** *Astragalus membranaceus*, hairy root, over-expression, astragalosides, gene expression

## Abstract

*Astragalus membranaceus* has been used as a highly popular Chinese herbal medicine for centuries. Triterpenoids, namely astragalosides I, II, III, and IV, represent the main active compounds in this plant species. Transcription factors have a powerful effect on metabolite biosynthesis in plants. We investigated the effect of the *Arabidopsis* MYB12, production of anthocyanin pigment 1 (PAP1), and maize leaf color (LC) transcription factors in regulating the synthesis of astragaloside metabolites in *A. membranaceus*. Overexpression of these transcription factors in hairy roots differentially up-regulated these active compounds. Specifically, the overexpression of LC resulted in the accumulation of astragalosides I–IV. The content of astragalosides I and IV were, in particular, more highly accumulated. Overexpression of MYB12 increased the accumulation of astragaloside I in transgenic hairy roots, followed by astragaloside IV, and overexpression of PAP1 resulted in the increased synthesis of astragalosides I and IV. In addition, we found that overexpression of PAP1 together with LC increased astragaloside III levels. At the transcriptional level, several key genes of the mevalonate biosynthetic pathway, especially *HMGR1*, *HMGR2*, and *HMGR3*, were up-regulated differentially in response to these transcription factors, resulting in astragaloside synthesis in the hairy roots of *A. membranaceus*. Overall, our results indicated that heterologous expression of *Arabidopsis* MYB12, PAP1, and maize LC differentially affected triterpenoids biosynthesis, leading to the increased biosynthesis of active compounds in *A. membranaceus*.

## 1. Introduction

*Astragalus membranaceus* (Fisch.) Bunge. has been used as a popular Chinese herbal medicine for many years. The dried roots of *A. membranaceus* are known as “Huang Qi” or “Radix astragali” and are used in many traditional Chinese herbal decoctions. In Europe, the United States, and Asian countries, Radix astragali are commonly used as dietary supplements and additives [1]. *A. membranaceus* has attracted the interest of many researchers due to their excellent pharmacological properties, including anticancer, anti-inflammatory, antioxidant and antiviral activities, immune enhancement, diabetic regulation and so on [2,3,4,5,6,7]. The astragalosides (AGs), including astragaloside I (AG I), astragaloside II (AG II), isoastragaloside II (IAG II), astragaloside III (AG III), astragaloside IV (AG IV) and cycloastragenol, are triterpenoid saponins and represent one of the main classes of active compounds in *A. membranaceus* [8,9]. Astragalosides occur uniquely in *Astragalus* species and exhibit multiple pharmacological activities that contribute to this herb’s wide range of applications [2,5,10,11,12,13,14]. 

Although AGs are important active ingredients, the total triterpene saponin content in *Astragalus* species is usually low, and this limited level of production restricts the pharmaceutical application of these herbal remedies [9,15]. However, researchers have attempted to increase the production of these active metabolites in *A. membranaceus* and other plants. For example, investigators have undertaken a feasibility study on treating the plants with elicitors, including methyl jasmonate (MeJA), salicylic acid, and acetylsalicylic acid, to promote AG production. Methyl jasmonate was shown to be the most effective at inducing AG production, with MeJA-treated plants exhibiting a 2.1-fold increase in AG concentration (5.5 ± 0.13 mg/g dry weight) than controls [16]. Moreover, Tuan et al. reported that the synthesis of AGs, calycosin, and calycosin-7-*O*-β-d-glucoside were efficiently up-regulated in MeJA-treated *A. membranaceus* hairy roots [17]. Yeast extract was also shown to facilitate AG production in *Agrobacterium*-mediated *A. membranaceus* hairy root cultures [18]. In other work, UV-B radiation was reported to promote AG production in *A. membranaceus* hairy roots [19]. However, the elicitor-induced production of metabolites has generally resulted in relatively low improvement and productivity [16,19]. 

Transcription factors are powerful regulators of metabolite biosynthesis in plants. It is well-recognized that transcription factors such as MYB, basic/helix-loop-helix (bHLH), and WD40 proteins can help researchers to engineer variations in metabolite biosynthesis [20,21]. The contents of flavonoids and anthocyanins, for example, have been reported to accumulate at high levels in many colorful tissues of fruits and vegetables, such as tomato flesh [22], potato tubers [23], bilberry [24], apple [25], strawberry [26], crabapple [27] and so on [20] under the manipulation of transcription factors. For example, the AP2/ERF family transcription factor PnERF1 was shown to positively regulate triterpenoid saponin biosynthesis in *Panax notoginseng* [28], and *PjERF1* from *Panax japonicus* was also reported to promote saponin biosynthesis in *PjERF1* overexpression cell lines [29]. Moreover, *Salvia miltiorrhiza* SmERF115 has been reported to be a positive regulator of phenolic acid biosynthesis in *S. miltiorrhiza* [30], while the R2R3-MYB transcription factors *SmMYB1* and *SmMYB97* have been found to improve the accumulation of phenolic acids, anthocyanins, and tanshinones in *S. miltiorrhiza* [31,32]. However, to the best of our knowledge, the metabolic engineering of metabolite biosynthesis in *A. membranaceus* using transcription factors has not yet been described thoroughly.

In this study, we used three transcription factors, the Arabidopsis transcription factor MYB12, Production of Anthocyanin Pigment1 (PAP1), and maize *Leaf color* (*Lc*), heterologously expressed in the *A. membranaceus* hairy root system, to evaluate whether they could be used to engineer AG metabolite biosynthesis. Our results indicated that MYB12, PAP1, and LC could differentially participate in triterpenoid biosynthesis. RT-PCR analysis revealed that overexpression of these three transcription factors induced the up-regulation of key structural genes in the AG biosynthetic pathway. This research provides useful information about the metabolic engineering of active compound production in the hairy roots of *A. membranaceus* using transcription factors. 

## 2. Results

### 2.1. Identification of A. membranaceus Hairy Root Overexpression Lines

**Figure 1:**
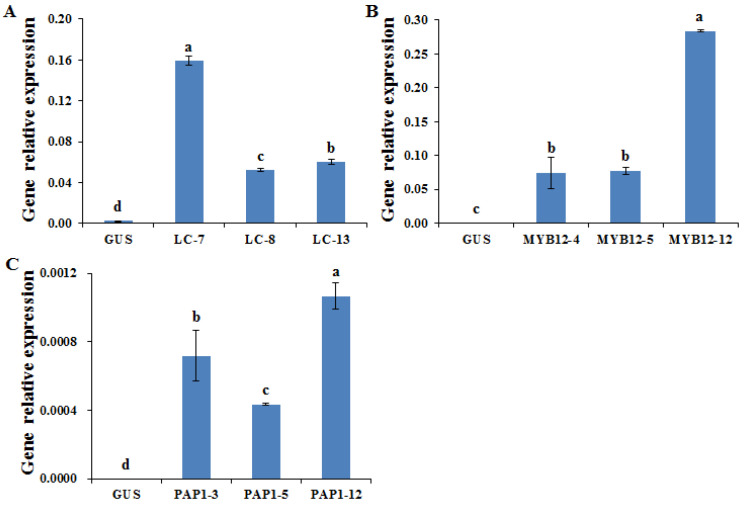
The expression level of *LC*-, *MYB12*-, and *PAP1*- overexpression hairy roots lines. (**A**) *LC*-overexpression hairy roots lines. (**B**) *MYB12*-overexpression hairy roots lines. (**C**) *PAP1*-overexpression hairy roots lines. GUS, GUS-overexpressing hairy root line (control). The height of each bar and the error bars show the mean and standard error, respectively, from three independent measurements. The different letters indicate significant differences at the 5% level by Duncan’s multiple range test.

After transformation and selection using an antibiotic medium, surviving hairy root lines were multi-cultured, and then the expression level of each transcription factor was confirmed by qRT-PCR analysis (Figure 1). According to qRT-PCR results, the expression levels of *ZmLc*, *AtMYB12*, and *AtPAP1* were detected higher in the transgenic lines than in that of the control GUS hairy roots lines. In particular, expression in the hairy root line LC-7 was 66.6-fold higher than in the control line, followed by LC-8 and LC-13, which displayed 21.8-fold and 25.2-fold higher expression than the control (Figure 1A). MYB12-12 and PAP1-12 showed at least 3.6-fold and 1.5-fold higher expression than the other two overexpression lines (Figure 1B,C). Finally, these higher overexpression lines of *ZmLc*, *AtMYB12*, and *AtPAP1* were selected for further analysis.

### 2.2. Transcription Levels of Biosynthetic Genes in AGs Biosynthetic Pathways

**Figure 2:**
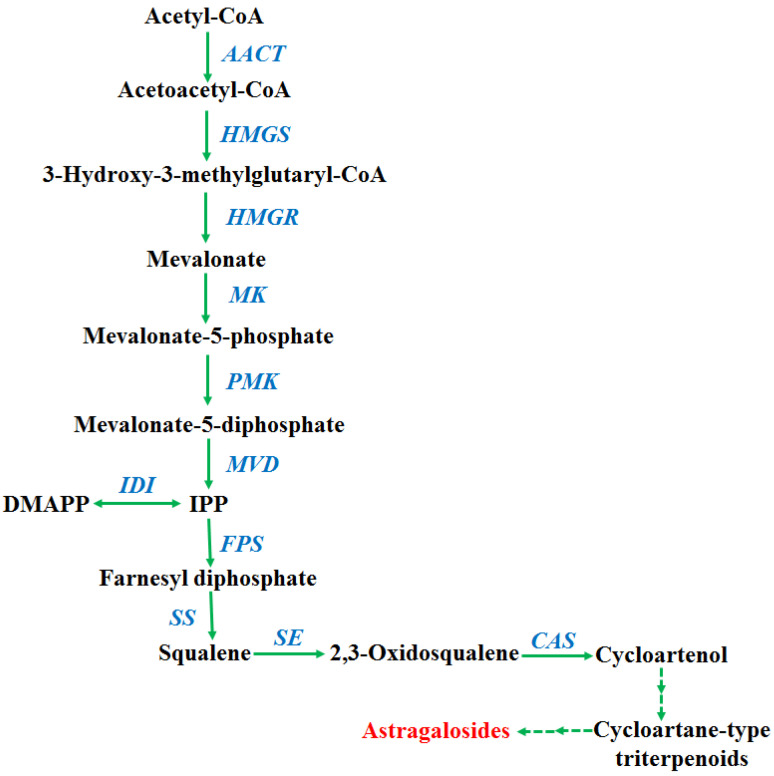
The proposed AGs biosynthetic pathway in *A. membranaceus*. AACT, acetoacetyl-CoA thiolase; HMGS, 3-hydroxy-3-methylglutaryl-CoA synthase; HMGR, 3-hydroxy-3-methylglutaryl-CoA reductase; PMK, phosphomevalonate kinase; MVD, mevalonate diphosphate decarboxylase; IDI, isopentenyl diphosphate isomerase; FPS, farnesyl diphosphate synthase; SS, squalene synthase; SE, squalene epoxidase; CAS, cycloartenol synthase.

**Figure 3:**
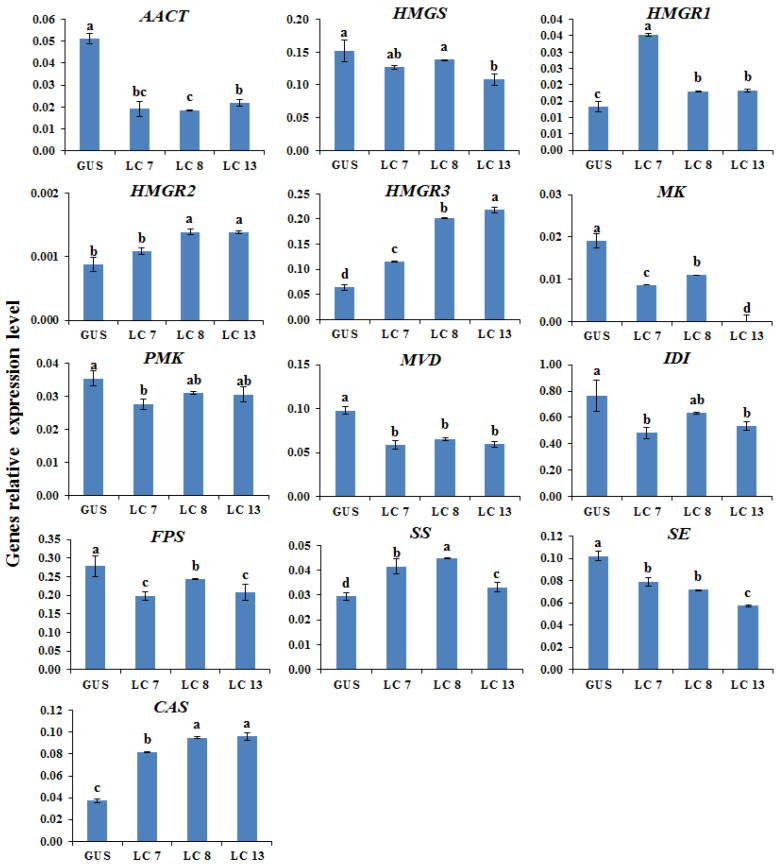
The relative expression levels of the key astragaloside biosynthetic genes in control and LC overexpression hairy root lines. GUS, GUS-overexpressing hairy root line (control). The height of each bar and the error bars show the mean and standard error, respectively, from three independent measurements. The different letters indicate significant differences at the 5% level by Duncan’s multiple range test.

**Figure 4:**
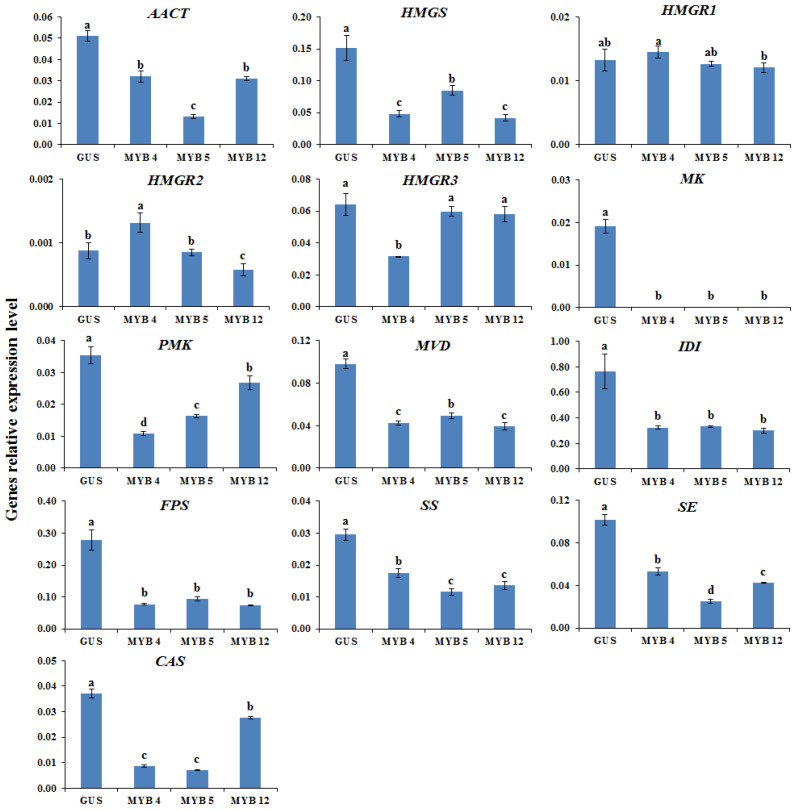
The relative expression levels of the key astragaloside biosynthetic genes in control and MYB12 overexpression hairy root lines. GUS, GUS-overexpressing hairy root line (control). The height of each bar and the error bars show the mean and standard error, respectively, from three independent measurements. The different letters indicate significant differences at the 5% level by Duncan’s multiple range test.

**Figure 5:**
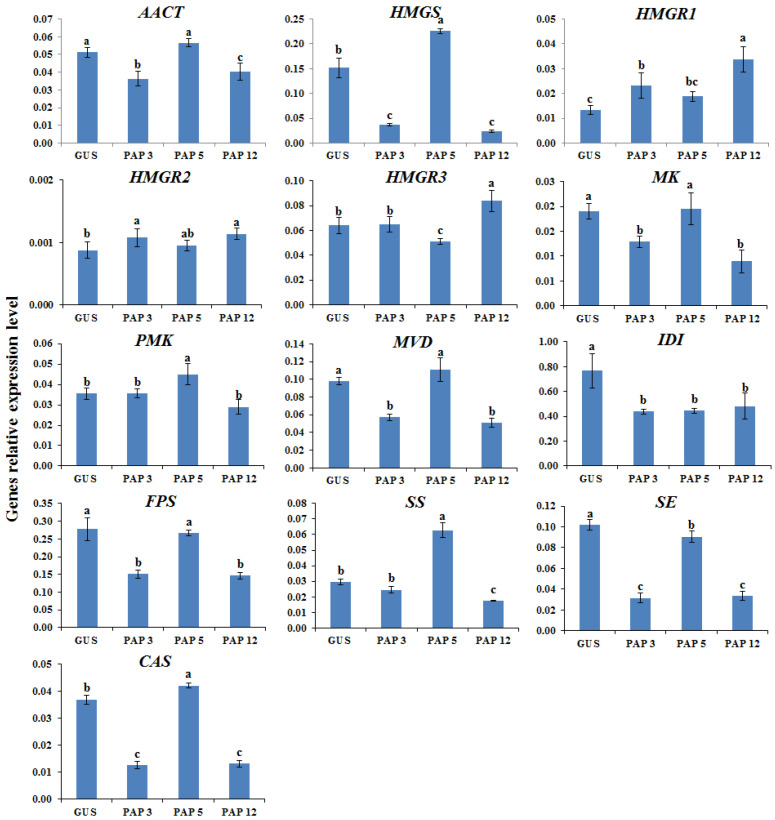
The relative expression levels of the key astragaloside biosynthetic genes in control and PAP1 overexpression hairy root lines. GUS, GUS-overexpressing hairy root line (control). The height of each bar and the error bars show the mean and standard error, respectively, from three independent measurements. The different letters indicate significant differences at the 5% level by Duncan’s multiple range test.

**Figure 6:**
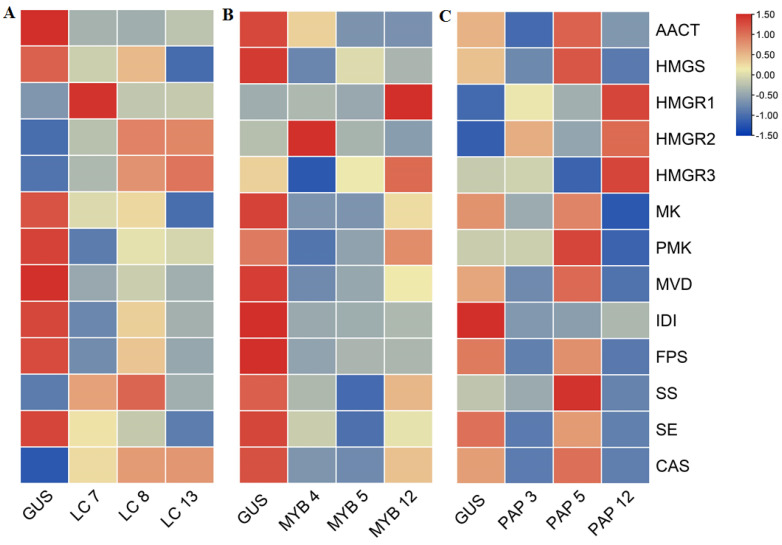
Heatmap analyses of AGs biosynthetic genes in *LC* (**A**), *MYB12* (**B**), and *PAP1* (**C**) overexpression hairy root lines. GUS, GUS-overexpressing hairy root line (control).

The proposed AGs biosynthetic pathway in *A. membranaceus* was shown in Figure 2 [33,34]. To clarify the molecular mechanism underlying the transcription factor manipulation, the expression of key genes involved in AGs biosynthetic pathway was determined by qRT-PCR. According to the results (Figure 3, Figure 4, Figure 5 and Figure 6), the biosynthetic genes in AGs biosynthetic pathway were differently regulated at the expressions level. In the *LC*-overexpression lines, the expression levels of *AmHMGR1*, *AmHMGR2*, *AmHMGR3*, *AmSS*, and *AmCAS* were up-regulated (Figure 3 and Figure 6). In particular, *AmHMGR* was reported as a key gene contributing to AG biosynthesis, the results indicated that *AmHMGRs* was mainly up-regulated in *LC*-overexpression lines. For the *Atmyb12* and *AtPAP1* overexpression lines, unexpectedly, the expression levels of *HMGR1*, *HMGR2*, or *HMGR3*, were found to be more stimulated than other genes in the AG biosynthetic pathway (Figure 4 and Figure 5). In all, the results indicated that the key genes involved in the AGs biosynthetic pathway were differentially response to the *LC*, *MYB12*, and *PAP1* transcription factors, which might result in the differential accumulation of metabolites.

### 2.3. Synthesis of AGs in Transgenic Hairy Roots of A. membranaceus

**Figure 7:**
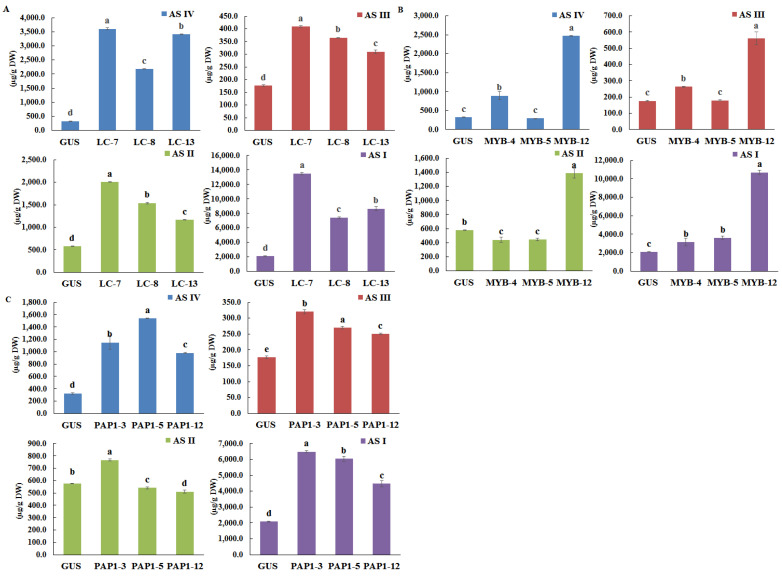
The content of four AGs in *LC* (**A**), *MYB12* (**B**), and *PAP1* (**C**) overexpression hairy root lines. GUS, GUS-overexpressing hairy root line (control). The height of each bar and the error bars show the mean and standard error, respectively, from three independent measurements. The different letters indicate significant differences at the 5% level by Duncan’s multiple range test.

The contents of AGs I, II, III, and IV in the transgenic hairy roots of *A. membranaceus* were then analyzed by HPLC (Figure 7 and Figure S1). The accumulation of most of the AGs increased in the three transgenic lines. Overexpression of LC resulted in an increased amount of all four AG compounds in the transgenic hairy roots. In particular, the content of AG IV was increased most highly in the lines *LC*-7, -8, and -13, which showed 11.1-, 6.7-, and 10.5-fold higher levels than the control, respectively. Next, AG I showed 6.5-, 3.6- and 4.1-fold higher levels than the control, respectively, in the three transgenic lines. AG II also exhibited a 2.0–3.5-fold increase in the LC transgenic hairy roots lines, followed by AG III, which displayed a 1.8–2.3-fold increase (Figure 7A). The four AGs were also up-regulated in the MYB12 transgenic hairy roots. The highest levels were detected in *MYB12*-12, where AG I, II, III, and IV exhibited 5.1-, 2.4-, 3.2- and 7.6-fold increases, respectively, compared with the control (Figure 7B), indicating that the higher rate of AG biosynthesis might be related to the considerable expression levels of the transcription factors. Overexpression of PAP1 showed a similar trend and contribution to AG synthesis; AGs I and IV exhibited a 2.1 and 4.7-fold increase, respectively, whereas AG II levels were only slightly increased in the PAP1 overexpression lines (Figure 7C).

## 3. Discussion

Hairy roots are commonly considered to be an alternative system in which to study metabolite biosynthesis in plants. The system offers a useful way of producing increased amounts of metabolites within a relatively short time frame when compared with traditional culture methods [35]. The *A. membranaceus* hairy root cultures system has been well established previously [17,36,37]. There were some reports about regulating AGs production in *A. membranaceus* hairy root cultures, and it has been reported that *A. rhizogenes*-mediated *A. membranaceus* 36-day-old hairy roots exhibited excellent AG production compared with 3-year-old field-grown roots [38]. The use of UV and MeJA as elicitors was shown to promote AG production in *A. membranaceus* hairy root cultures [16,19]. Moreover, combining deacetylation biocatalysis with the elicitation of immobilized *Penicillium canescens* (IPC) treatment, enhanced the content of AG IV was enhanced by 14.59-fold when compared with the control in *A. membranaceus* hairy root cultures [39]. However, as far as we know, little information is available about metabolic genetic engineering in *A. membranaceus* using transcription factors via the hairy root system.

Arabidopsis transcription factors MYB12, PAP1, and LC are three previously characterized flavonoid and/or anthocyanin-related transcription factors [40,41,42,43]. *AtMYB12* is considered to be a flavonol-specific activator of flavonoid biosynthesis in *Arabidopsis* [42]. The MYB transcription factor *AtPAP1* has been reported to be a positive anthocyanin and flavonoid regulator [44]. The ectopic expression of *PAP1* was found to improve the production of anthocyanins and antioxidant activity in Ginseng hairy roots [41]. Anthocyanin accumulation was also enhanced by the overexpression of *PAP1/AtMYB75* in the transgenic hop (*Humulus lupulus* L.), canola (*Brassica napus*), and so on [45,46]. *Leaf color* (*Lc*) belongs to the maize R gene family and was the first basic/helix-loop-helix (bHLH) transcription factor found in maize, *Lc* acts in the regulation of anthocyanin synthesis in maize [47], and heterologous expression of maize *Lc* can allow the engineering of flavonoid and/or anthocyanin biosynthesis in many plants, such as Arabidopsis and tobacco [43,48], lisianthus and a regal pelargonium cultivar [49], rice [50], sweet potato [51], and golden root [40]. Park et al., reported that *ZmLc* and *AtPAP1* can be used as positive regulators in the metabolic engineering of flavonoid biosynthesis in *S*. *baicalensis*, resulting in 3- and 5-fold higher total flavonoids, respectively, than the control [40]. In this study, our results also indicated that the overexpression of *ZmLc*, *AtMYB12* and *AtPAP1* efficiently up-regulated AG biosynthesis in *A. membranaceus*, especially, AG I and AG IV were considered as common targets and significantly higher accumulation in overexpression hairy roots lines.

It has been shown in many plant species that transcription factors regulate the transcript levels of structural genes and contribute to the accumulation of metabolites [20,21]. Moreover, it has been reported that the heterologous expression of IbMYB1a positively controls the expression of multiple anthocyanin biosynthetic genes, and results in enhanced-anthocyanin synthesis in tobacco [52]. Multiple omics analyses have revealed that the main biosynthetic genes *PAL*, *CHS*, *CHI*, *F3H*, and *FLS* are upregulated in the flavonoid biosynthesis pathway and resulted in the accumulation of flavonol and flavonoid derivatives in *AtMYB12* overexpressed tomato [53]. Anthocyanin accumulation was enhanced by the overexpression of *PAP1/AtMYB75* in transgenic canola (*Brassica napus*) and hop (*Humulus lupulus* L.), by regulating the transcription level of flavonoid biosynthesis-related structural genes [46]. However, these regulated targets have not always been the same in different plants. For example, although *AtMYB12* and *PAP1* were overexpressed in buckwheat hairy root, only the *AtMYB12* overexpressed lines resulted in rutin enhancement through the up-regulation of *PAL*, *C4H*, *4CL*, *CHS*, *CHI*, *F3H*, *F3′H*, and *FLS* genes in the flavonoid biosynthesis pathway [54], while, ectopic expression of *PAP1* indeed enhanced the expression of various genes involved in the phenylpropanoid biosynthetic pathways (24 genes), and flavonoid biosynthetic pathways (17 genes) in ginseng (*Panax ginseng* C.A. Meyer) hairy roots [41]. 

The key genes in the AG biosynthesis pathway in *A. membranaceus* have previously been reported. [18,36,55]. For example, HMGR is a key rate-limiting enzyme for triterpenoid biosynthesis and the expression of the *HMGR* gene is directly related to triterpenoid biosynthesis [16,56,57,58,59]. The production of phytosterols and triterpenoids was enhanced by overexpressing *Panax ginseng* HMGR (*PgHMGR*) in *Platycodon grandiflorum* [58]. Jiao et al. [16] reported that a significant increase in the accumulation of total AGs was detected by UV-B elicitation; however, even among the major genes, *AACT* to *CAS* of the AG biosynthesis pathway, only the higher expression of *HMGR* gene was induced by UV-B elicitation [16]. In this research, the relative expression levels of key genes related to AG biosynthetic pathway were detected. Interestingly, our results were similar to those found previously, which also demonstrated that *HMGRs* played a key target gene in *A. membranaceus* AGs biosynthesis and responded to the regulation of *ZmLc*, *AtMYB12*, and *AtPAP1* [19]. 

According to previous research, the cytochrome P450 monooxygenase (P450) and the glycosyltransferases (GTs) play important roles in the triterpenoid pathway, mainly creating structural diversity of triterpenoids in plants [60,61,62]. However, even triterpenoids-related P450 or GTs has been reported in some plant, such as *Arabidopsis thaliana*, *Panax ginseng*, *Saponaria vaccaria* [63,64,65], GTs and P450 genes are also speculated to be involved in regulating AGs biosynthesis, but it remains unclear how P450s and GTs play a role in oxidation and glycosylation during AGs biosynthesis [16,34].

## 4. Materials and Methods

### 4.1. Plant Materials

*Astragalus membranaceus* seeds were purchased from Wofeng Chinese traditional Medicine Co., Ltd. (Hebei, China), and were authenticated by Prof. Sangun Park. The *Astragalus membranaceus* sterilized seeds were obtained similarly as described before Li et al. [66]. The surface-sterilized procedure was accomplished with 70% ethanol and 4% (*v*/*v*) bleach solution, followed by rinsing several times in sterile water. The sterilized seeds were cultured on 1/2 MS medium in a growth chamber with a photoperiod of 16 h light/8 h dark at 24 °C. *A. membranaceus* 20 day old seedlings were used as the explant materials for hairy roots induction [17].

### 4.2. Vector Construction and Generation of Transgenic Hairy Roots

The vector construction method has been described as previously reported [40]. Briefly, TFs *AtMYB12*, *ZmLc*, and *AtPAP1* (GenBank accession ID: AAC83586, DQ414252.1, and NM_104541.3) were cloned, and the overexpression vector construction was accomplished through BP clonase II and LR clonase (Invitrogen, Carlsbad, CA, USA) according to the manufacturer’s instructions. Finally, these genes were successfully inserted into the overexpression vector pK7FWG2 (Invitrogen), expression clones pK7FWG2-*ZmLc* and pK7FWG2-*AtPAP1* were then transformed into *Agrobacterium rhizogenes* strain R1000 by electroporation transformation. The GUS gene, which overexpresses beta-glucuronidase, was also constructed by the same method as control. The *A. membranaceus* hairy roots transformants were generated using the *A. rhizogenes*-mediated method [36]. Briefly, hairy roots were induced from leaves of *A. membranaceus* infected by *A. rhizogenes* R1000 [17,36]. Transformants were selected on 1/2MS media with kanamycin (50 mg/L) and cefotaxime (250 mg/L) at 25 °C in dark conditions. The survived hairy root lines were sub-cultured in 30 mL of 1/2 MS liquid medium on a shaker (100 rpm) in dark conditions for 3 weeks. Hairy root samples were harvested, and then the overexpression lines for each TFs were confirmed by qRT-PCR analysis. 

### 4.3. Quantitative Real-Time PCR

Total RNA isolation and cDNA synthesis was finished as followed [66]: Total RNA was isolated from samples using the RNeasy Plant Mini Kit (Qiagen, Valencia, CA, USA). For cDNA synthesis, 1 μg of total RNA was reverse-transcribed using the Superscript II First Strand SynthesisKit (Invitrogen, Carlsbad, CA, USA) and an oligo (dT)20 primer. The expression of key genes involved in AGs biosynthetic pathway was determined by qRT-PCR. The primer sets used for qRT-PCR was listed as the same as references [34,40,54]. The relative expression levels of genes were normalized to that of an internal reference 18S gene (ZX0811025). The qRT-PCR method was as same as the reference [54]. Results were calculated as the mean of three replicates. 

### 4.4. HPLC Analysis for AGs

The control and confirmed overexpression hairy root lines were freeze-dried and ground into a fine powder. Samples (100 mg) were extracted with MeOH (1.5 mL) by sonicator for 60 min, then the supernatant was filtered by 0.45 μm PTFE syringe filter (Advantec DISMIC-13HP; Toyo Roshi Kaisha, Ltd., Tokyo, Japan) for HPLC analysis. The C_18_ column (250 × 4.6 mm, 5 μm; RStech; Daejeon, Korea) was employed for HPLC analysis. The HPLC was equipped with evaporating light scattering detection (ELSD). Astragalosides (AGs) chemical compounds including astragaloside I (AG I), astragaloside II (AG II), isoastragaloside II (IAG II), astragaloside III (AG III), astragaloside IV (AG IV) were obtained from ChromaDex (Irvine, CA, USA). The HPLC-ELSD analysis method for Astragalosides (AGs) was established in the previous study [17,36]. The concentration of AGs was determined using the external standardization method. All samples were analyzed in triplicate.

### 4.5. Statistical Analysis

All the experiments were independently conducted in triplicate, and the results for gene expression and metabolites content were shown as the mean ± standard error (SE). TB tools software was used for heatmap analysis. The ANOVA analysis was performed using SPSS version 22 (SPSS Inc, Chicago, IL, USA). The significant differences between sample groups were conducted using Duncan’s Multiple Range Test. Values with different letters mean significantly different between groups (*p* < 0.05).

## 5. Conclusions

Taken together, the hairy root system is considered to be a useful and suitable tool for the genetic engineering of secondary metabolites enhancement. The efficient regulation of secondary metabolite production may be achieved by investigating the relevant transcription factors, especially in medicinal plants. In this study, we provide useful information about the use of transcription factors in the genetic engineering of AG biosynthesis in *A. membranaceus*, and show this to be a useful model for further exploring and investigating the function of transcription factors in triterpenoid accumulation in *A. membranaceus* and other plants.

## Data Availability

Data reported are available in the Supplementary Materials.

## Supplementary Materials

The following supporting information can be downloaded at: https://www.mdpi.com/article/10.3390/plants11141897/s1. Figure S1: Representative HPLC chromatograms of AGs analysis. (A) AGs standards; (B) Control, GUS-overexpressing hairy root line; (C) LC- overexpression hairy root line; (D) comparation of AGs standards (red) and sample (blue). Peaks: 1, astragaloside IV; 2, astragaloside III; 3, astragaloside II; 4, astragaloside I.

## References

[1] Chu C., Qi L.-W., Liu E.-H., Li B., Gao W., Li P. (2010). Radix Astragali (Astragalus): Latest advancements and trends in chemistry, analysis, pharmacology and pharmacokinetics. Curr. Org. Chem..

[2] Kathy K.A., Han Q.-B., Joshua K.K. (2016). *Astragalus membranaceus*: A review of its protection against inflammation and gastrointestinal cancers. Am. J. Chin. Med..

[3] Sheik A., Kwanwoo K., Ganji Lakshmi V., Lee H., Kim S., Kim E., Shin J.-Y., Oh S.Y., Huh Y.S. (2021). The anti-cancerous activity of adaptogenic herb *Astragalus membranaceus*. Phytomedicine.

[4] Liu A., Yu J., Ji H., Zhang H., Zhang Y., Liu H. (2018). Extraction of a novel cold-water-soluble polysaccharide from *Astragalus membranaceus* and its antitumor and immunological activities. Molecules.

[5] Shan H., Zheng X., Li M. (2019). The effects of *Astragalus membranaceus* active extracts on autophagy-related diseases. Int. J. Mol. Sci..

[6] Elabd H., Wang H.-P., Shaheen A., Yao H., Abbass A. (2016). *Astragalus membranaceus* (AM) enhances growth performance and antioxidant stress profiles in bluegill sunfish (*Lepomis macrochirus*). Fish Physiol. Biochem..

[7] Zheng Y., Ren W., Zhang L., Zhang Y., Liu D., Liu Y. (2020). A review of the pharmacological action of *Astragalus* Polysaccharide. Front. Pharmacol..

[8] Li Y., Guo S., Zhu Y., Yan H., Qian D.-W., Wang H.-Q., Yu J.-Q., Duan J.-A. (2019). Comparative analysis of twenty-five compounds in different parts of *Astragalus membranaceus* var. *mongholicus* and *Astragalus membranaceus* by UPLC-MS/MS. J. Pharm. Anal..

[9] Napolitano A., Akay S., Mari A., Bedir E., Pizza C., Piacente S. (2013). An analytical approach based on ESI-MS, LC–MS and PCA for the quali–quantitative analysis of cycloartane derivatives in *Astragalus* spp.. J. Pharm. Biomed. Anal..

[10] Guo T., Liu Z.-L., Zhao Q., Zhao Z.-M., Liu C.-H. (2018). A combination of astragaloside I, levistilide A and calycosin exerts anti-liver fibrosis effects in vitro and in vivo. Acta Pharmacol. Sin..

[11] Wang Z.-F., Ma D.-G., Zhu Z., Mu Y.-P., Yang Y.-Y., Feng L., Yang H., Liang J.-Q., Liu Y.-Y., Liu L. (2018). Astragaloside IV inhibits pathological functions of gastric cancer-associated fibroblasts. World J. Gastroenterol..

[12] Fu Y., Cai J., Xi M., He Y., Zhao Y., Zheng Y., Zhang Y., Xi J., He Y. (2020). Neuroprotection effect of Astragaloside IV from 2-DG-induced endoplasmic reticulum stress. Oxid. Med. Cell. Longev..

[13] Hao M., Liu Y., Chen P., Jiang H., Kuang H.-Y. (2018). Astragaloside IV protects RGC-5 cells against oxidative stress. Neural Regen. Res..

[14] Indu P., Arunagirinathan N., Rameshkumar Marimuthu R., Sangeetha K., Divyadarshini A., Rajarajan S. (2020). Antiviral activity of astragaloside II, astragaloside III and astragaloside IV compounds against dengue virus: Computational docking and in vitro studies. Microb. Pathog..

[15] Song J.-Z., Mo S.-F., Yip Y.-K., Qiao C.-F., Han Q.-B., Xu H.-X. (2007). Development of microwave assisted extraction for the simultaneous determination of isoflavonoids and saponins in *Radix Astragali* by high performance liquid chromatography. J. Sep. Sci..

[16] Jiao J., Gai Q.-Y., Wang W., Luo M., Zu Y.-G., Fu Y.-J., Ma W. (2016). Enhanced astragaloside production and transcriptional responses of biosynthetic genes in *Astragalus membranaceus* hairy root cultures by elicitation with methyl jasmonate. Biochem. Eng. J..

[17] Tuan P.A., Chung E., Thwe A.A., Li X., Kim Y.B., Mariadhas V.A., Al-Dhabi N.A., Lee J.H., Park S.U. (2015). Transcriptional profiling and molecular characterization of astragalosides, calycosin, and calycosin-7-O-β-D-glucoside biosynthesis in the hairy roots of *Astragalus membranaceus* in response to methyl jasmonate. J. Agric. Food Chem..

[18] Park Y.J., Kim J.K., Park S.U. (2021). Yeast extract improved biosynthesis of astragalosides in hairy root cultures of Astragalus membranaceus. Prep. Biochem. Biotechnol..

[19] Gai Q.-Y., Jiao J., Luo M., Wang W., Zhao C.-J., Fu Y.-J., Ma W. (2016). UV elicitation for promoting astragaloside production in *Astragalus membranaceus* hairy root cultures with transcriptional expression of biosynthetic genes. Ind. Crops Prod..

[20] Naing A.H., Kim C.K. (2018). Roles of R2R3-MYB transcription factors in transcriptional regulation of anthocyanin biosynthesis in horticultural plants. Plant Mol. Biol..

[21] Allan A.C., Hellens R.P., Laing W.A. (2008). MYB transcription factors that colour our fruit. Trends Plant Sci..

[22] Sun C., Deng L., Du M., Zhao J., Chen Q., Huang T., Jiang H., Li C.-B., Li C. (2020). A transcriptional network promotes anthocyanin biosynthesis in tomato flesh. Mol. Plant.

[23] Zhang H., Yang B., Liu J., Guo D., Hou J., Chen S., Song B., Xie C. (2017). Analysis of structural genes and key transcription factors related to anthocyanin biosynthesis in potato tubers. Sci. Hortic..

[24] Katja K., Declan J.L., Nick W.A., Nelli M., Tony M., Andrew C.A., Bilal M.A., Hely H., Richard V.E., Laura J. (2021). MYBA and MYBPA transcription factors co-regulate anthocyanin biosynthesis in blue-coloured berries. New Phytol..

[25] Ban Y., Honda C., Hatsuyama Y., Igarashi M., Bessho H., Moriguchi T. (2007). Isolation and functional analysis of a MYB transcription factor gene that is a key regulator for the development of red coloration in apple skin. Plant Cell Physiol..

[26] Zhang Z., Shi Y., Ma Y., Yang X., Yin X., Zhang Y., Xiao Y., Liu W., Li Y., Li S. (2020). The strawberry transcription factor FaRAV1 positively regulates anthocyanin accumulation by activation of FaMYB10 and anthocyanin pathway genes. Plant Biotechnol. J..

[27] Tian J., Zhang J., Han Z.-Y., Song T.-T., Li J.-Y., Wang Y.-R., Yao Y.-C. (2017). McMYB12 Transcription factors co-regulate proanthocyanidin and anthocyanin biosynthesis in *Malus* crabapple. Sci. Rep..

[28] Deng B., Huang Z., Ge F., Liu D., Lu R., Chen C. (2017). An AP2/ERF Family Transcription factor PnERF1 raised the biosynthesis of saponins in *Panax notoginseng*. J. Plant Growth Regul..

[29] Chen Q., Yu Y., Zhang X., Zhao R., Zhang J., Liu D., Cui X., Ge F. (2021). The transcription factor PjERF1 enhances the biosynthesis of triterpenoid saponins in *Panax japonicus*. Plant Biotechnol. Rep..

[30] Sun M., Shi M., Wang Y., Huang Q., Yuan T., Wang Q., Wang C., Zhou W., Kai G. (2019). The biosynthesis of phenolic acids is positively regulated by the JA-responsive transcription factor ERF115 in *Salvia miltiorrhiza*. J. Exp. Bot..

[31] Zhou W., Shi M., Deng C., Lu S., Huang F., Wang Y., Kai G. (2021). The methyl jasmonate-responsive transcription factor SmMYB1 promotes phenolic acid biosynthesis in *Salvia miltiorrhiza*. Hortic. Res..

[32] Li L., Wang D., Zhou L., Yu X., Yan X., Zhang Q., Li B., Liu Y., Zhou W., Cao X. (2020). JA-responsive transcription factor SmMYB97 promotes phenolic acid and tanshinone accumulation in *Salvia miltiorrhiza*. J. Agric. Food Chem..

[33] Lichtenthaler H.K., Rohmer M., Schwender J. (1997). Two independent biochemical pathways for isopentenyl diphosphate and isoprenoid biosynthesis in higher plants. Physiol. Plant..

[34] Kim Y.B., Thwe A.A., Li X., Tuan P.A., Lee S., Lee J.W., Arasu M.V., Al-Dhabi N.A., Park S.U. (2014). Accumulation of astragalosides and related gene expression in different organs of *Astragalus membranaceus* Bge. var *mongholicus* (Bge.). Molecules.

[35] Shi M., Liao P., Nile S.H., Georgiev M.I., Kai G. (2021). Biotechnological Exploration of Transformed Root Culture for Value-Added Products. Trends Biotechnol..

[36] Park Y.J., Thwe A.A., Li X., Kim Y.J., Kim J.K., Arasu M.V., Al-Dhabi N.A., Park S.U. (2015). Triterpene and flavonoid biosynthesis and metabolic profiling of hairy roots, adventitious roots, and seedling roots of *Astragalus membranaceus*. J. Agric. Food Chem..

[37] Hirotani M., Zhou Y., Lui H., Furuya T. (1994). Astragalosides from hairy root cultures of *Astragalus membranaceus*. Phytochemistry.

[38] Jiao J., Gai Q.-Y., Fu Y.-J., Ma W., Yao L.-P., Feng C., Xia X.-X. (2015). Optimization of Astragalus membranaceus hairy roots induction and culture conditions for augmentation production of astragalosides. Plant Cell Tissue Organ Cult..

[39] Gai Q.-Y., Jiao J., Luo M., Wang W., Yao L.-P., Fu Y.-J. (2017). Deacetylation biocatalysis and elicitation by immobilized *Penicillium canescens* in *Astragalus membranaceus* hairy root cultures: Towards the enhanced and sustainable production of astragaloside IV. Plant Biotechnol. J..

[40] Park C.H., Xu H., Yeo H.J., Park Y.E., Hwang G.-S., Park N.I., Park S.U. (2021). Enhancement of the flavone contents of Scutellaria baicalensis hairy roots via metabolic engineering using maize Lc and Arabidopsis PAP1 transcription factors. Metab. Eng..

[41] Jin S., Hyun T.K. (2020). Ectopic Expression of production of Anthocyanin Pigment 1 (PAP1) improves the antioxidant and anti-melanogenic properties of Ginseng (*Panax ginseng* C.A. Meyer) hairy roots. Antioxidants.

[42] Mehrtens F., Kranz H., Bednarek P., Weisshaar B. (2005). The Arabidopsis transcription factor MYB12 is a flavonol-specific regulator of phenylpropanoid biosynthesis. Plant Physiol..

[43] Lloyd A.M., Walbot V., Davis R.W. (1992). Arabidopsis and Nicotiana anthocyanin production activated by maize regulators R and C1. Science.

[44] Borevitz J.O., Xia Y., Blount J., Dixon R.A., Lamb C. (2000). Activation tagging identifies a conserved MYB regulator of phenylpropanoid biosynthesis. Plant Cell.

[45] Gatica-Arias A., Farag M.A., Stanke M., Matoušek J., Wessjohann L., Weber G. (2012). Flavonoid production in transgenic hop (*Humulus lupulus* L.) altered by PAP1/MYB75 from *Arabidopsis thaliana* L. Plant Cell Rep..

[46] Li X., Gao M.-J., Pan H.-Y., Cui D.-J., Gruber M.Y. (2010). Purple Canola: *Arabidopsis* PAP1 increases antioxidants and phenolics in *Brassica napus* leaves. J. Agric. Food Chem..

[47] Ludwig S.R., Habera L.F., Dellaporta S.L., Wessler S.R. (1989). Lc, a member of the maize R gene family responsible for tissue-specific anthocyanin production, encodes a protein similar to transcriptional activators and contains the myc-homology region. Proc. Natl. Acad. Sci. USA.

[48] Huang Z.-A., Zhao T., Wang N., Zheng S.-s. (2016). Ectopic expression of Lc differentially regulated anthocyanin biosynthesis in the floral parts of tobacco (*Nicotiana tobacum* L.) plants. Bot. Stud..

[49] Bradley J.M., Deroles S.C., Boase M.R., Bloor S., Swinny E., Davies K.M. (1999). Variation in the ability of the maize Lc regulatory gene to upregulate flavonoid biosynthesis in heterologous systems. Plant Sci..

[50] Song Y.E., Wang X., Shen Z.W., Xu Y., Li J.Y. (2013). Expressing the maize anthocyanin regulatory gene Lc increased flavonoid content in the seed of white pericarp rice and purple pericarp rice. Russ. J. Genet..

[51] Wang H., Yang J., Zhang M., Fan W., Firon N., Pattanaik S., Yuan L., Zhang P. (2016). Altered phenylpropanoid metabolism in the maize Lc-expressed sweet potato (*Ipomoea batatas*) affects storage root development. Sci. Rep..

[52] An C.H., Lee K.W., Lee S.H., Jeong Y.J., Woo S.G., Chun H., Park Y.I., Kwak S.S., Kim C.Y. (2015). Heterologous expression of IbMYB1a by different promoters exhibits different patterns of anthocyanin accumulation in tobacco. Plant Physiol. Biochem..

[53] Meng X., Zhao X., Ding X., Li Y., Cao G., Chu Z., Su X., Liu Y., Chen X., Guo J. (2020). Integrated functional omics analysis of flavonoid-related metabolism in AtMYB12 transcript factor overexpressed tomato. J. Agric. Food Chem..

[54] Nam Il P., Xiaohua L., Aye Aye T., Sook Young L., Su Gwan K., Qi W., Sang Un P. (2011). Enhancement of rutin in Fagopyrum esculentum hairy root cultures by the *Arabidopsis* transcription factor AtMYB12. Biotechnol. Lett..

[55] Jun L., Yuka H.-L., Matthew D.D., Qianjin F., Judith R.R., Zhipeng Q., David L.A. (2017). Long read reference genome-free reconstruction of a full-length transcriptome from *Astragalus membranaceus* reveals transcript variants involved in bioactive compound biosynthesis. Cell Discov..

[56] Chappell J., Wolf F., Proulx J., Cuellar R., Saunders C. (1995). Is the reaction catalyzed by 3-hydroxy-3-methylglutaryl coenzyme A reductase a rate-limiting step for isoprenoid biosynthesis in plants?. Plant Physiol..

[57] Hey S.J., Powers S.J., Beale M.H., Hawkins N.D., Ward J.L., Halford N.G. (2006). Enhanced seed phytosterol accumulation through expression of a modified HMG-CoA reductase. Plant Biotechnol. J..

[58] Kim Y.-K., Kim J.K., Kim Y.B., Lee S., Kim S.-U., Park S.U. (2013). Enhanced accumulation of phytosterol and triterpene in hairy root cultures of *Platycodon grandiflorum* by overexpression of *Panax ginseng* 3-hydroxy-3-methylglutaryl-coenzyme A reductase. J. Agric. Food Chem..

[59] Bach T.J. (1995). Some new aspects of isoprenoid biosynthesis in plants—A review. Lipids.

[60] Karel M., Jacob P., Dieter B., Philipp A., René C., Sven S., Tessa M., Jan M., Prashant D.S., Laurens P. (2017). The ancient CYP716 family is a major contributor to the diversification of eudicot triterpenoid biosynthesis. Nat. Commun..

[61] Satoru S., Kazuki S. (2011). Triterpenoid biosynthesis and engineering in plants. Front. Plant Sci..

[62] Harrison D.M. (1985). The biosynthesis of triterpenoids and steroids. Nat. Prod. Rep..

[63] Suzanne P., Birger Lindberg M., Søren B. (2003). On the origin of family 1 plant glycosyltransferases. Phytochemistry.

[64] Xu W., Bak S., Decker A., Paquette S.M., Feyereisen R., Galbraith D.W. (2001). Microarray-based analysis of gene expression in very large gene families: The cytochrome P450 gene superfamily of *Arabidopsis thaliana*. Gene.

[65] Yao C., Hanbing L., Xuejiao T., Zaimin L., Xin Z., Dalong L., Xinmei J., Xihong Y. (2020). Identification and analysis of CYP450 and UGT supergene family members from the transcriptome of *Aralia elata* (Miq.) seem reveal candidate genes for triterpenoid saponin biosynthesis. BMC Plant Biol..

[66] Li X., Thwe A.A., Park N.I., Suzuki T., Kim S.J., Park S.U. (2012). Accumulation of phenylpropanoids and correlated gene expression during the development of tartary buckwheat sprouts. J. Agric. Food Chem..

