# Supplementary material for: Heterologous Expression of Three Transcription Factors Differently Regulated Astragalosides Metabolic Biosynthesis in Astragalus membranaceus Hairy Roots
Source: Plants (Basel). 2022 Jul 21;11(14):1897. doi: 10.3390/plants11141897 (PMC9315567; doi:10.3390/plants11141897)
Supplement: Supplementary file 1 [file plants-11-01897-s001.zip › plants-1743668-supplementary.pdf]

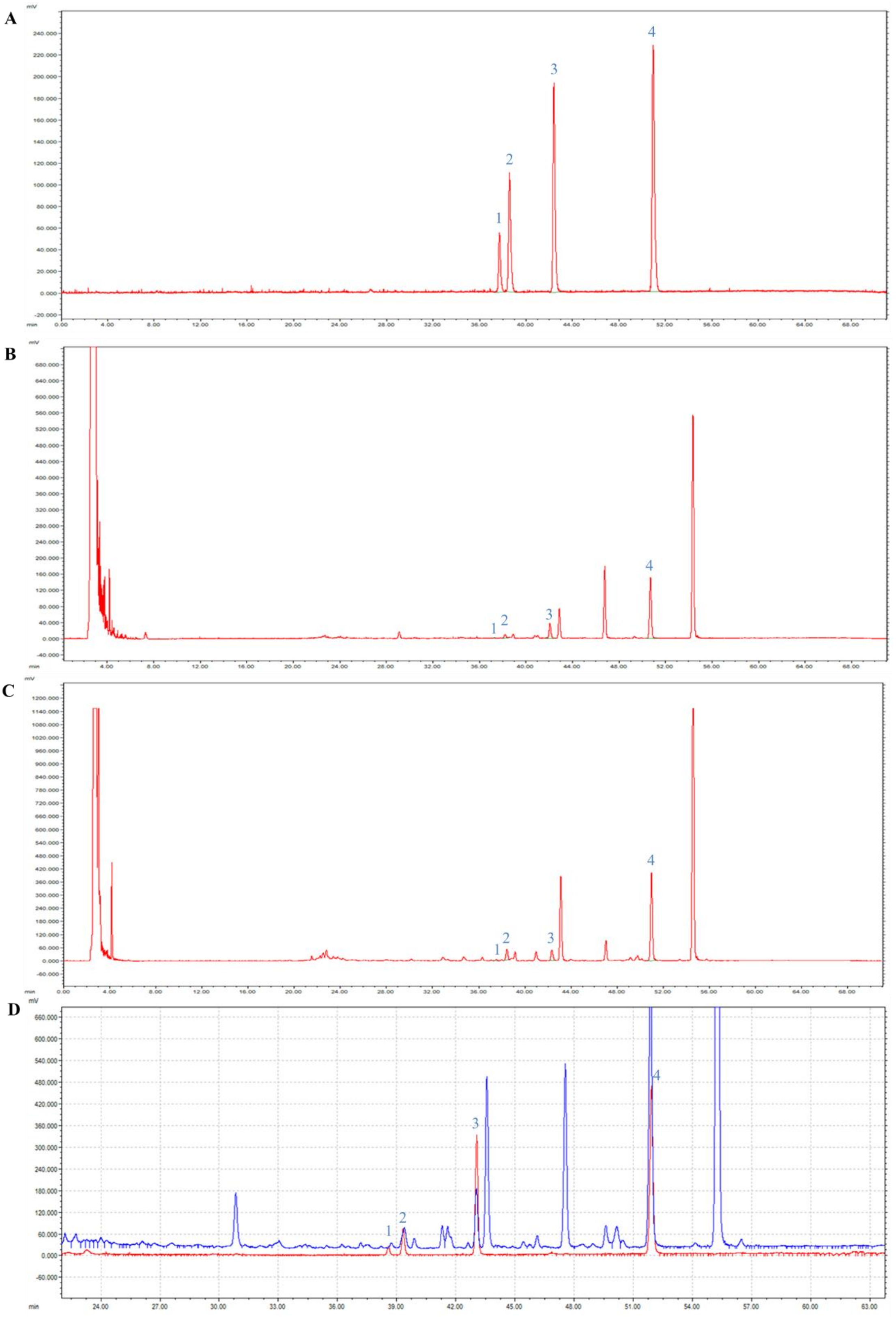

Figure S1. Representative HPLC chromatograms of AGs analysis. (A) AGs standards; (B) Control, GUS-overexpressing hairy root line; (C) *LC*- overexpression hairy root line; (D) comparation of AGs standards (red) and sample (blue). Peaks: 1, astragaloside IV; 2, astragaloside III; 3, astragaloside II; 4, astragaloside I.
